# The longitudinal validity of proxy-reported CHU9D

**DOI:** 10.1007/s11136-021-02774-9

**Published:** 2021-02-13

**Authors:** Rasmus Trap Wolf, Julie Ratcliffe, Gang Chen, Pia Jeppesen

**Affiliations:** 1 Danish Centre for Health Economics, Department of Public Health, University of Southern Denmark, Odense, Denmark; 2 Child and Adolescent Mental Health Centre, Mental Health Services-Capital Region of Denmark, Odense, Denmark; 3 College of Nursing and Health Sciences, Flinders University, Bedford Park, Australia; 4 Centre for Health Economics, Monash University, Melbourne, Australia; 5 Department of Clinical Medicine, Faculty of Health and Medical Sciences, University of Copenhagen, Copenhagen, Denmark

**Keywords:** CHU9D, Health-related quality of life, Health state utility value, Children, Adolescents, Mental health, Preference weights

## Abstract

**Objectives:**

The Child Health Utility 9D (CHU9D) currently represents the only preference-based health-related quality-of-life instrument designed exclusively from its inception for application with children. The objective of this study was to examine the construct validity and responsiveness of the proxy-reported (parent) CHU9D in a mental health setting using utility weights derived from an adult and adolescent population, respectively.

**Methods:**

The discriminant validity and convergent validity were examined using the mental health-specific ‘The Strengths and Difficulties Questionnaire’ (SDQ) and the generic KIDSCREEN-27. Responsiveness was assessed by examining the floor-ceiling effects, the magnitude of change over time, and the ability to differentiate between improvement and no improvement.

**Results:**

The study included 396 children with mental health problems. CHU9D showed good construct validity, with correlation coefficients ranging between 0.329 and 0.571 for SDQ Impact score and KIDSCREEN-27 Psychological Well-being. CHU9D was able to distinguish between groups of children with different levels of mental health problems (*p* < 0.001). The absolute magnitudes of the group mean differences were larger using adolescent weights. No evidence of a floor/ceiling effect was found at the baseline. A standardized response mean of 0.634–0.654 was found for the children who experienced clinically significant improvements. CHU9D was able to discriminate between children who experienced positive and no health improvements (*p* < 0.001).

**Conclusion:**

This study provides the first evidence on responsiveness for CHU9D in a mental health context. The findings demonstrate that CHU9D is an appropriate HRQOL measure for use in mental health trials. Furthermore, the results show that the preference weights generated from an adolescent population resulted in the larger mean differences between groups.

**Supplementary Information:**

The online version contains supplementary material available at 10.1007/s11136-021-02774-9.

## Introduction

Evidence of the cost-effectiveness of new and emerging interventions is commonly used across health systems to assist policy makers in the allocation of scarce health care resources. In order to conduct cost–utility analysis, the most prevalent form of economic evaluation, a preference-based measure of health-related quality of life (HRQOL) is widely used to facilitate the calculation of quality-adjusted life years (QALYs), a generic measure of effectiveness [1]. Preference-based HRQOL measures provide a single-summary score which makes it a useful outcome measure also across multiple settings.

During the last decade, there has been an increased focus on preference-based HRQOL instruments that aim to measure children’s utility. The guidelines for estimating QALYs in youth populations are, however, still unclear [2]. A recent review, therefore, warrants further empirical evidence on the valuation of youth-specific preference-based measures [3]. A review identifies nine preference-based HRQOL instruments that have been used in paediatric populations [4]. Of these the Child Health Utility 9D (CHU9D) is the only one designed exclusively from its inception for application with young people [5]. The remaining instruments were not originally developed for children and adolescents, and others represent different forms of adoptions from measures designed for adults.

CHU9D has been demonstrated in several studies to have good content, face, and construct validity for young people in the age-group 7-17 years [6–13]. Furber & Segal [14] examined face validity, practicality, internal consistency, and convergent validity of CHU9D in a population of clients in South Australian child and adolescent mental health services. The authors concluded that their initial validation of CHU9D showed promising results, but that there was a need for further validation including a general need for validation of responsiveness, which their cross-sectional design was unable to capture. Responsiveness is critical for a preference-based HRQOL instrument, as its suitability for application in economic evaluation depends on its capacity to reliably detect changes in HRQOL due to the introduction of new interventions. To the best of our knowledge, the responsiveness of CHU9D has not yet been examined.

There exist two main scoring algorithms for deriving utilities from CHU9D, one based on an adult population [15] and one based on an adolescent population [16]. The choice of whose values to use in an economic evaluation can have important policy implications due to the potential impact this has on the QALY estimates and thus on the final incremental cost-effectiveness ratio. The choice could especially be important in interventions aiming to improve mental health, as Ratcliffe and colleagues found that adolescents placed more weight upon impairments in CHU9D dimensions related to mental health (sad, worried, annoyed) than adults [8].

The main objective of this study is to examine the construct validity and responsiveness of the proxy-reported (parent) CHU9D in a mental health setting. This will be the first study to examine the validity of CHU9D a longitudinal design, and the first study to examine responsiveness of CHU9D in a mental health context. Furthermore, the examination of construct validity will add to the evidence from Furber & Segal [14], on the appropriateness of using CHU9D in a mental health setting by examining a larger population and having comparison with both a mental health-specific measure (the Strengths and Difficulties Questionnaire, SDQ) and a generic HRQOL measure (KIDSCREEN-27). A second objective is to examine whether the utility weights derived from the adult population or the utility weights derived from the adolescent population demonstrate differences in validity and responsiveness in this context.

## Methods

### Participants

Participants in this study were part of the Mind My Mind trial (Trial ID: NCT03535805). The details of the trial are described elsewhere [17, 18]. Briefly, the methods and study population are as follows.

The trial is designed to evaluate the effectiveness and cost-effectiveness of a new transdiagnostic modular cognitive and behavioral treatment versus treatment-as-usual for school-aged children with emotional and/or behavioral disturbances. The program comprises 9–13 weekly individual sessions targeting anxiety, depression and/or behavioral problems. The management-as-usual varied, as the children could receive anonymous counseling, pedagogical advice, network meetings, educational support, or psychological treatment of various kinds, either publicly or privately funded, or no further treatment.

The trial was advertised for professionals and parents in the community by use of pamphlets and intra-/internet, and the recruitment was based on parent’s initiative to seek help in collaboration with professionals such as the schoolteacher, nurse or psychologist. The inclusion procedure included minimum scores derived from the SDQ as reported by the parents and a clinical interview with a psychologist. The minimum scores from the SDQ follow a screening algorithm designed to identify children with mental health problems in the need of an intervention, this is further described elsewhere [19]. To be included in the trial, the child had to have a primary problem that falls within the domains of anxiety, depressive symptoms or behavioral problems, according to the classification by the psychologist conducting the interview. Children with prior mental disorder diagnosis and children with an indication of severe mental disorders (e.g., signs of a full syndrome of ADHD and autism spectrum disorder) were excluded. Children with parents that did not understand and speak Danish sufficiently to participate in the trial were excluded. Based on sample size calculation and a pilot trial a total of 396 children aged 6–15 from four Danish municipalities (Helsingør, Holstebro, Næstved and Vordingborg) across the country were included and randomized on an individual level to the intervention or treatment-as-usual in the four community mental health care settings.

### Measures

Data were collected via an online platform at both baseline and end-of-treatment 18 weeks later. Children and parents as proxies for the children completed the Danish versions of SDQ, KIDSCREEN-27 (KIDSCREEN) and CHU9D at baseline and end-of-treatment. This study solely focuses on parental responses since the SDQ was not reported by children younger than 11 years (which accounts for 48% of the study population). All three questionnaires were in Danish using validated translations [7, 20, 21].

#### The strengths and difficulties questionnaire

The SDQ is a widely used and well validated questionnaire aiming to assess children’s mental health problems in both clinical samples and in general population [22–25]. The SDQ contains 25 items, which cover five subscales relating to the children’s emotional problems, peer problems, behavioural problems, hyperactivity and pro-social behaviour. Responses to the subscales on emotional problems, peer problems, behavioural problems, and hyperactivity can be used to calculate a total difficulties score (SDQ-TD). Each subscale score ranges from 0–10, implying that the total difficulties score ranges from 0 to 40 [26]. An extended version of the SDQ includes an impact assessment to evaluate how much the identified mental difficulties interfere with the child’s everyday life. An impact score (SDQ-I) is calculated from five items; whether the difficulties upsets or distresses the child and how much the difficulties interfere with home life, friendships, classroom learning, and leisure activities. Each item is scored on a scale from 0 to 2. To score 1 or 2, the interference from the difficulties in that domain must be assessed to either “quite a lot” or “a great deal” [24]. The impact score is the primary outcome of the Mind My Mind Trial. When completing the SDQ parents were asked to respond as a proxy for their child based upon the preceding six months at baseline, and the preceding month at end-of-treatment.

#### KIDSCREEN

KIDSCREEN is a 27-item generic measure of HRQOL and well-being. A total of 13 European countries were included in the cross-cultural harmonization and development of the measure. Several studies have found it to be valid and reliable in children with and without chronic health conditions, demonstrating adequate psychometric properties [20–27]. KIDSCREEN measures HRQOL and well-being across five domains: Physical Well-being, Psychological Well-being, Autonomy & Parents, Peers & Social Support and School Environment. Item responses are based on a five-point Likert scale and T scores for each domain are computed with a mean of 50 and standard deviation of 10, whereby higher scores indicate better HRQOL [28]. KIDSCREEN domain scores do not allow for the calculation of a global HRQOL score. When completing the KIDSCREEN, the parents were asked to respond as a proxy for their child based upon the last week.

#### Child health utility 9D

CHU9D is a generic preference-based HRQOL measure designed specifically for use in an economic evaluation of health care interventions in children and adolescents. CHU9D has nine items with five levels of severity representing nine dimensions of HRQOL: Worried, Sad, Pain, Tired, Annoyed, Schoolwork/homework, Sleep, Daily routine, and Activities. The responses to the nine items can be converted to utilities, on the 0–1 dead–full health QALY scale, using preference-based scoring algorithms. In this study, two separate scoring algorithms were applied. The original algorithm is based on the standard gamble method of health state valuation and the preferences of an adult general population in the United Kingdom (*N* = 300). This algorithm generates utility scores ranging from 0.3261 (pit-state) to 1 (perfect health) [10]. A newer algorithm is based on best–worst scaling methods and the preferences of adolescent Australians aged 11–17 from the general population (*N* = 1982) and a smaller sample (*N* = 152) time-trade-off experiment with young adults to anchor the tariffs. This algorithm generates utility scores ranging from − 0.1059 (pit-state) to 1 (perfect health) [16]. Danish-specific preference weights are not yet available. When completing CHU9D, the parents were asked to respond as a proxy for their child based upon their HRQOL on the present day.

#### Conceptual overlaps

Table 1 provides a simplified overview of the conceptual overlaps between the three different measures. We categorized the items and the subscales of the three instruments into seven dimensions of quality of life based on direct comparisons of the content, even though the content and concepts are not likely to be independent of each other. Table 1 provides information on which measures we hypothesize CHU9D to be closest related to. Thus, CHU9D has the largest conceptual overlap with SDQ-I followed by SDQ-TD and KIDSCREEN’s Physical Well-being and Psychological Well-being. Contrary, there is no clear conceptual overlap between CHU9D and the KIDSCREEN Social Support & Peers measure.

**Table 1:** Simplified conceptual overlap between measures by quality of life dimensions

	Quality of life dimensions						
	Mental health	Physical health	Sleep	Parental relation	Social relations	School	Activities/daily routine
Child health utility 9D	X	X	X			X	X
SDQ total difficulties score	X				X		
SDQ impact score	X			X	X	X	X
KIDSCREEN physical well-being		X					X
KIDSCREEN psychological well-being	X						X
KIDSCREEN autonomy & parent relation				X			
KIDSCREEN social support & peers					X		
KIDSCREEN school environment						X	

### Psychometric analyses

#### Construct validity

To assess construct validity, the baseline data were used, and the discriminant validity and convergent validity were examined.

Discriminant validity was assessed by testing whether CHU9D can discriminate between groups defined by the SDQ-TD, the SDQ-I and the KIDSCREEN Psychological well-being score. The entire sample in this study exhibited some degree of mental health problems distributed on a continuum, and it was, therefore, not possible to define clearly distinguishable categories. Instead, we assessed whether CHU9D could distinguish between groups of children with different levels of problems using percentiles as cut-off values on the SDQ-TD, SDQ-I, and KIDSCREEN Psychological Well-being score. We focused on these scores as mental health problems are expected to have the largest impact on HRQOL. The study sample is divided into three groups: the children with the 25% lowest scores (low), the 25% highest scores (high) and the 50% in between (medium). Statistical differences were tested using Kruskal–Wallis test due to non-normality of the utility distributions (tested using the Shapiro–Francia test), and the magnitude of mean difference was assessed based on a minimally important difference (MID) of 0.03 [29] as no formal MID is available for CHU9D.

Convergent validity was assessed using Spearman rank correlation coefficients. Correlation between CHU9D, the SDQ-TD, the SDQ-I and the KIDSCREEN scores was assessed. Based on Table 1, we hypothesized moderate correlations between CHU9D and the SDQ-TD and SDQ-I scores, KIDSCREEN Psychological Well-being and Physical Well-being. For the other KIDSCREEN scores, we hypothesized a low but positive correlation, as higher scores in these conceptually less overlapping dimensions of HRQOL would to some degree still be expected to correlate with higher CHU9D utility scores. A complete correlation matrix at the dimension/item level for CHU9D and each of the SDQ and KIDSCREEN scores and items is available in the appendix. Following established guidelines, the following categories for Spearman rank correlations are used: ≥ 0.5, strong; ≥ 0.3 to < 0.5, moderate; and < 0.3, weak [30].

#### Responsiveness

To assess responsiveness, the floor and ceiling effects were first examined; next, the magnitude of change over time and the ability to differentiate between improvement and no improvement were investigated.

Floor or ceiling effects (i.e., more than 15% of respondents scored the lowest or highest possible score) affect the ability of the measure to detect deterioration or improvements in health, respectively [31]. For CHU9D, we hypothesized a low percentage at the floor and ceiling at baseline, but we expected a higher percentage at the ceiling at follow-up, given that an effective intervention should improve the mental health of the respondents randomized to intervention. The floor and ceiling effects on SDQ and KIDSCREEN scores are used as reference values for examining CHU9D.

The magnitude of change in scores from baseline to end-of-treatment was assessed using the standardized response mean (SRM) statistic. The following categories for SRM are used: < 0.2 small; 0.5, moderate; and > 0.8, large [30]. We first report the SRM for the whole sample. To study the responsiveness, we identified sub-groups of children whose mental health condition had improved according to the standardized measures SDQ and KIDSCREEN. Children who had improved at least 1 point on the SDQ-I were examined, as this is considered a minimum clinically important difference [32]. For these groups of children with improved mental health, we hypothesized that CHU9D demonstrates a change in the same direction as SDQ and KIDSCREEN Psychological Well-being scale. Given that the latter two scales are more specific to the intervention, it was expected that larger effects would be found relative to CHU9D. The SRMs from the SDQ and KIDSCREEN scales are presented as reference values.

The mean change in CHU9D score for the children with improved mental health was estimated and compared with the mean changes for the children whose condition did not improve or got worse. Due to non-normality of the utility distributions, statistical differences were tested using Mann–Whitney test. The interpretation of the magnitude of mean difference was again based on a MID of 0.03.

## Results

The characteristics of the participants and the baseline scores are presented in Table 2. Using the Mann–Whitney test/Kruskal–Wallis test, no associations (*p* > 0.1) between age, gender or parent’s education and CHU9D score were found regardless of the scoring algorithm applied. A total of 57 (14%) were lost to follow-up and were not included in the analyses of responsiveness. Logistic regression models found that the father’s highest education being bachelor/diploma was the only background variable that statistically significant (*p* > 0.05) predicted the loss to follow-up.

**Table 2:** Characteristics and baseline scores for participants

	*n*	Mean (SD)/%
Age, mean (standard deviation)	396	10.7 (2.36)
Gender, %		
Boy	206	52.0%
Girl	190	48.0%
Parent registered as informant, %		
Mother	342	86.4%
Father	54	13.6%
Mothers’ highest achieved education, %		
Basic school (0–10 years)	26	6.6%
High school/vocational education	112	28.3%
Bachelor/diploma degree	216	54.5%
Master/PhD	42	10.6%
Fathers’ highest achieved education, %		
Basic school (0–10 years)	60	15.3%
High school/vocational education	166	42.2%
Bachelor/Diploma degree	120	30.5%
Master/PhD	47	12.0%
Baseline scores, mean (standard deviation)		
CHU9D adult weights	396	0.804 (0.121)
CHU9D adolescent weights	396	0.629 (0.214)
SDQ Total difficulties score (SDQ-TD)	395	16.06 (5.36)
SDQ Impact Score (SDQ-I)	395	4.16 (2.39)
KIDSCREEN Physical Well-being	396	44.40 (10.2)
KIDSCREEN Psychological Well-being	396	43.95 (9.12)
KIDSCREEN Autonomy & Parent Relation	394	46.93 (9.05)
KIDSCREEN Social Support & Peers	395	43.75 (10.8)
KIDSCREEN School Environment	394	46.16 (9.02)

*CHU9D* Child Health Utility 9D, *SDQ* The Strength and Difficulties Questionnaire, *KIDSCREEN* KIDSCREEN-27

### Construct validity

#### Discriminant validity

CHU9D performed well in discriminating between the groups scoring low, medium, and high on the three mental health-related scales regardless of the scoring algorithm applied. Table 3 shows that the group mean differences on CHU9D utility scores were all statistically significant and larger than the commonly used MID value of 0.03 [29]. The absolute magnitudes on the group mean differences were larger when using the adolescent weights than the adult weights.

**Table 3:** Discriminant validity

		CHU9D adult weights, mean (SD)	CHU9D adolescent weights, mean (SD)
SDQ Total difficulties score (SDQ-TS)	Low (0–12) *n* = 106	0.857 (0.095)	0.725 (0.175)
	Medium (13–19) *n* = 184	0.796 (0.119)	0.615 (0.212)
	High (20–40) *n* = 105	0.765 (0.129)	0.558 (0.220)
	P level*	< 0.001	< 0.001
SDQ impact score (SDQ-I)	Low (0–2) *n* = 100	0.858 (0.093)	0.729 (0.172)
	Medium (3–5) *n* = 186	0.807 (0.114)	0.631 (0.203)
	High (6–10) *n* = 109	0.750 (0.133)	0.535 (0.225)
	P level*	< 0.001	< 0.001
KIDSCREEN psychological well-being	High (44.4–100) *n* = 95	0.890 (0.076)	0.789 (0.134)
	Medium (34–44.3) *n* = 198	0.812 (0.099)	0.640 (0.184)
	Low (0–33) *n* = 103	0.710 (0.128)	0.460 (0.204)
	P level*	< 0.001	< 0.001

*Kruskal–Wallis test

*CHU9D* Child Health Utility 9D, *SDQ* The Strength and Difficulties Questionnaire, *KIDSCREEN* KIDSCREEN-27, *SD* Standard Deviation

#### Convergent validity

In assessing the convergent validity, the correlations between CHU9D and SDQ-TS, SDQ-I and the KIDSCREEN scores were calculated; the correlation coefficients were similar regardless of CHU9D weights. Moderate correlations between CHU9D score and the SDQ-I and the KIDSCREEN Physical Well-being, respectively, were found as hypothesized. For KIDSCREEN Psychological Well-being a strong correlation was evident with CHU9D, whilst a weak (but close to moderate) correlation was found for SDQ-TD with CHU9D. For the remaining KIDSCREEN scores, weak correlations were found in the hypothesized direction, as higher KIDSREEN score associated with higher CHU9D utility score. Consistent with the overview of overlapping concepts presented in Table 1, a stronger correlation with CHU9D was found for KIDSCRREN School Environment compared to Autonomy & Parent Relation and Support & Peers (Table 4).

**Table 4:** Spearman rank correlation coefficients for SDQ, KIDSCREEN and CHU9D utility scores

	CHU9D adult weights	CHU9D adolescent weights
SDQ total difficulties score (SDQ-TD)	− 0.285	− 0.294
SDQ impact score (SDQ-I)	− 0.330	− 0.329
KIDSCREEN physical Well-being	0.438	0.408
KIDSCREEN psychological well-being	0.565	0.571
KIDSCREEN autonomy & parent relation	0.197	0.200
KIDSCREEN social support & Peers	0.217	0.187
KIDSCREEN school environment	0.266	0.260

All *P* < 0.001

CHU9D: Child Health Utility 9D. SDQ: The Strength and Difficulties Questionnaire. KIDSCREEN: KIDSCREEN-27

Despite not measuring the exact same concepts and the different time recall periods for each instrument, the specific CHU9D dimensions/items correlated with specific items of the SDQ and one of the KIDSCREEN scales, in accordance with our hypotheses based on item content. E.g., moderate correlations were found between CHU9D item “sad” and SDQ item “Often unhappy” and CHU9D item “worried” and KIDSCREEN Psychological Well-being. A complete correlation matrix on item and scale levels between CHU9D and the SDQ and KIDSCREEN is presented in the Appendix.

### Responsiveness

#### Floor and ceiling effect

When examining CHU9D utility scores at baseline and end-of-treatment, no evidence of a floor effect was found. None of the children in our study sample were reported as being in the worst health state at either time point. A ceiling effect was evident for CHU9D at end-of-treatment since 54 children (16%) reported as having no impairments (full health) in all nine dimensions. There was no ceiling effect at baseline since only 12 children (3%) reported to be in full health. In comparison, the same tendency was found for the SDQ-I, where 30 children (8%) had the lowest possible score (no impact) at baseline and 105 children (31%) had it at end-of-treatment. In contrast, both the SDQ-TD and the KIDSCREEN scores exhibited neither floor nor ceiling effects (between 0 and 4%).

#### Standard response mean

For CHU9D using adult weights, a mean improvement of 0.055 with a standard deviation of 0.121 was found resulting in a SRM of 0.452. In contrast, for CHU9D the adolescent weights generated a mean improvement of 0.102 with a standard deviation of 0.220, equivalent to a SRM of 0.462. In comparison SRMs of between 0.458 and 0.767 were found for the mental health-specific scores; SDQ-TD, SDQ-I and KIDSCREEN Psychological Well-being. For the non-mental health KIDSCREEN scores, we found SRMs between 0.131 and 0.323.

A total of 233 children obtained a clinical improvement of more than 1 point on the SDQ-I (which is deemed the minimum important difference [32]). This group exhibited a moderate SRM of 0.634 (adult weights) and 0.654 (adolescent weights).

#### Discriminant validity

CHU9D was able to discriminate between the group of children that experienced an improvement and the group of children that did not on both the SDQ-TD, SDQ-I and KIDSCREEN Well-being score. The differences in mean change were statistically significant and larger than the MID of 0.03. As seen in Table 5 the mean difference between the groups was considerably larger when using the adolescent weights compared to when using the adult weights.

**Table 5:** Mean change in CHU9D utility scores for those who improved, or did not improve, post-intervention and the mean difference between these two groups

Change in	Change in CHU9D adult weights, mean (*n*)	Difference, mean (95% CI)	Change in CHU9D adolescent weights, mean (*n*)	Difference, mean (95% CI)
**SDQ Total difficulties score (SDQ-TD)**				0.105*** (0.055;0.155)
≥ 0 *n* = 105	0.017 (105)	0.055***	0.030 (105)	
< 0 (improved score) *n* = 233	0.072 (233)	(0.028;0.083)	0.135 (233)	
**SDQ Impact Score (SDQ-I)**				0.111*** (0.061;0.160)
≥ 0 *n* = 105	0.015 (105)	0.059***	0.026 (105)	
< 0 (improved score) *n* = 233	0.074 (233)	(0.032;0.086)	0.137 (233)	
**KIDSCREEN Physical Well-being**				0.168*** (0.117;0.219)
< 0 *n* = 86	− 0.018 (86)	0.098***	− 0.023 (86)	
≥ 0 (improved score) *n* = 253	0.080 (253)	(0.070;0.126)	0.144 (253)	

Mann–Whitney test *P > 0.05 **P > 0.01 ***P > 0.001

*CHU9D* Child Health Utility 9D, *SDQ* The Strength and Difficulties Questionnaire, *KIDSCREEN* KIDSCREEN-27. *CI* confidence interval

## Discussion

In examining the construct validity of CHU9D in a mental health setting, this study has demonstrated that CHU9D is capable of discriminating between groups with different severity of mental health problems. In all cases, the mean difference between the groups was higher than the MID of 0.03. The utilities derived using the adolescent scoring algorithm did, however, result in substantial larger mean differences between groups. The average mean difference between the low-medium–high groups was 0.115 across the three measures using adolescent weights, while it was 0.063 when using the utilities derived from the adult weights. There can be different explanations for the differences. There are substantial methodological differences between the two sets of preference weights including the country, the sample sizes and the elicitation techniques. The differences found is, however, likely to be reflective of the relatively stronger weight attached to mental health impairments in the adolescent scoring algorithm in comparison with the adult scoring algorithm [10, 16]. A difference has also been found for CHU9D when comparing adolescent and adult preferences in the same country using the same methods for elicitation [8]. When used in a cost–utility analysis the choice of preference weights difference is likely to have a substantial impact on the incremental cost-effectiveness ratio (ICER). Future cost–utility analysis involving interventions for children with mental health problems could examine the impact of the choice of preference weights on the results of a CUA by conducting their analyses using both value sets.

For convergent validity, similar results were evident regardless of the weights used to derive CHU9D utilities. CHU9D showed the hypothesized correlations with all measures except with the SDQ-TD. Here a correlation just below 0.3 was found and thereby categorized as weak. The weak correlation could be due to the differences in the scope of CHU9D and SDQ-TD. CHU9D aims to capture the impact of mental and other health-related problems of the child, and the SDQ-TD aims to measure the symptoms of the mental health problems. To further analyze the correlation we compared our item correlations with those found by Furber and Segal [14]. In their study, they highlighted five correlations at the dimension/item-level which they argued have a clear conceptual overlap. Moderate correlations were found for three of them and weak correlations for two. In comparison, this study found moderate correlations for four of them and a weak correlation for one (correlations are marked in Appendix Table B). Furber and Segal [14] furthermore found correlations between CHU9D utility (adult weights) and SDQ items above 0.2 for 11 of the 20 items, which makes the Total difficulties score, whereas this study found correlations of 0.1 or lower for the same correlations (correlation are marked in Appendix Table B). In examining these 11 items in our cohort, on average only 12% of respondents indicated that their child was in the worst category. For six of the 11 items, *well behaved, one good friend, often fights and bullies, often lies/cheats, picked on/bullied,* and *steals* we found that there were less than 10% that responded in the worst category. These findings suggest lower levels of social and behavioral problems in this specific population, and, therefore, less convergence on these domains with a generic measure of HRQOL Combined with the convergent validity of the other measures the above findings make the weak correlation between CHU9D and SDQ-TD less of a concern in relation to convergent validity.

There was neither floor nor ceiling effects of CHU9D. Although 16% of the children reported full health in the follow-up, it should reflect the fact that after the intervention, their HRQOL improved. The findings from SDQ-I showed that an even higher percentage reported no impact on the daily life from the mental health problems at follow-up.

The analyses of SRM showed acceptable responsiveness of CHU9D regardless of the weights used to derive utilities. In the analyses of change in mean utility, CHU9D was capable of distinguishing between the group of children whose mental health improved and those who did not. Using CHU9D adolescent scoring algorithm, we found that the magnitude of the mean differences was considerably larger compared to when using the adult weights. These results again point towards the choice of utility weights is likely to have a great impact on the ICER in a cost–utility analysis.

The difference in recall time adopted in different questionnaires may influence the validation analysis of CHU9D. However, all questionnaires were completed online at the same time, which could possibly minimize the impact of the time perspective differences.

It is beyond the scope of the present study to describe the group differences in change scores. A cost–utility analysis of the intervention using CHU9D will be conducted later and published in a separate article.

This study provides a broad validation for the use of CHU9D in mental health settings as the participants consist of children with a broad range of mental health problems, ranging from internalizing to externalizing problems and combinations. The results are, however, limited in their generalizability due to the lack of participants with severe mental disorders. E.g., commonly used preference-based HRQOL instruments in adult populations have been shown to be less appropriate in trials with schizophrenia patients [28]. The cross-sectional findings by Furber and Segal [14] in a population that include severe mental disorders does, however, indicate CHU9D is also appropriate for use in such populations. A previous study has examined the validity of other preference-based HRQOL in a youth population suffering from depressive conditions. A number of the instruments, including non-pediatric, showed good construct validity and responsiveness in the study [34]. Future studies should examine if other non-pediatric preference-based HRQOL instruments show similar good construct validity and responsiveness also in other mental disorders and in younger populations.

In this study we examine the proxy-reported (parent) version of CHU9D, future studies should also examine the longitudinal validity of the self-reported version of CHU9D.

## Conclusions

The findings from this study demonstrate that the proxy-reported (parent) CHU9D is an appropriate preference-based HRQOL measure for use in mental health trials. The inclusion of CHU9D will enable a cost–utility analysis of interventions aiming to improve child and adolescent mental health, and thereby provide valuable evidence for health care resource allocation and decision-making.

The results showed that the preference weights generated from an adolescent population resulted in the larger mean differences between groups with different severity of mental health problems, and between the children that measured with SDQ and KIDSCREEN improved their mental health and those who did not. This finding suggests that the choice of preference weights could have a substantial impact on the results when used in a cost–utility analysis in a mental health setting.

## Supplementary Information

Below is the link to the electronic supplementary material.

Supplementary file1 (PDF 231 KB)

## Data Availability

The data utilized in the current study are defined as sensitive personal data, and cannot be shared publicly due to existing data protection laws in Denmark, and imposed by the Danish Data Protection Agency.
