# Supplementary material for: The longitudinal validity of proxy-reported CHU9D
Source: Qual Life Res. 2021 Feb 13;30(6):1747–56. doi: 10.1007/s11136-021-02774-9 (PMC8178153; doi:10.1007/s11136-021-02774-9)
Supplement: Supplementary file 1 — Supplementary file1 (PDF 231 KB) [file 11136_2021_2774_MOESM1_ESM.pdf]

Wolf, RT., Ratcliffe, J., Chen, G., Jeppesen, P. The longitudinal validity of proxy-reported CHU9D.

Appendix

Table A: Spearman rank correlation coefficients for CHU9D items, SDQ Total difficulties score, SDQ Impact score and KIDSCREEN-27

	Worried	Sad	Pain	Tired	Annoyed	Schoolwork / homework	Sleep	Daily Routine	Activities
SDQ Total difficulties score	0.053	0.256***	0.083	0.036	0.195***	0.243***	0.018	0.251***	0.230***
SDQ Impact Score	0.183***	0.308***	0.074	0.113*	0.160**	0.248***	0.020	0.282***	0.346***
KIDSCREEN Physical Well-being	-0.266***	-0.329***	-0.251***	-0.283***	-0.103*	-0.172***	-0.157**	-0.257***	-0.436***
KIDSCREEN Psychological Well-being	-0.439***	-0.541***	-0.186***	-0.309***	-0.222***	-0.265***	-0.220***	-0.334***	-0.430***
KIDSCREEN Autonomy & Parent Relation	-0.004	-0.103*	-0.052	0.049	-0.177***	-0.153**	-0.079	-0.227***	-0.079
KIDSCREEN Social Support & Peers	-0.150**	-0.173***	-0.079	-0.056	-0.028	-0.121*	-0.046	-0.117*	-0.316***
KIDSCREEN School Environment	-0.114*	-168***	0.039	-0.012	-0.118*	-0.458***	-0.080	-0.199***	-0.297***

Note: * P<0.05 **P<0.01 ***P<0.001

Wolf, RT., Ratcliffe, J., Chen, G., Jeppesen, P. The longitudinal validity of proxy-reported CHU9D.

Table B: Spearman rank correlation coefficients for CHU9D items, SDQ scales and SDQ items.

	Worried	Sad	Pain	Tired	Annoyed	Schoolwork /homework	Sleep	Daily Routine	Activities	**CHU9D adult weights**	**CHU9D adolescent weights**
**Total difficulties**	0.053	0.256***	0.083	0.036	0.195***	0.243***	0.018	0.251***	0.230***	-0.285***	-0.294***
**Emotion scale**	0.435***	0.342***	0.248***	0.134**	0.008	0.051	0.237***	0.143**	0.271***	-0.348***	-0.365***
**Conduct scale**	-0.265***	0.044	-0.046	-0.036	0.321***	0.074	-0.074	0.236***	0.036	-0.105*	-0.082
**Hyper scale**	-0.099*	0.074	-0.022	-0.026	0.170***	0.265***	-0.041	0.209***	0.096*	-0.131**	-0.149**
**Peer scale**	0.049	0.115*	-0.003	0.001	-0.037	0.152**	-0.070	-0.006	0.125*	-0.068	-0.070
**Prosocial scale #**	0.160**	-0.005	0.108*	0.091	-0.219***	-0.105*	0.109*	-0.132**	-0.057	0.036	0.032
Considerate #	0.222***	-0.038	0.078	0.064	-0.246***	-0.061	0.064	-0.084	-0.023	0.041	0.016
Restless	-0.051	0.017	-0.021	-0.017	0.079	0.108*	0.019	0.166***	0.001	*-0.045*	-0.078
Sickness	0.286***	0.248***	0.282***	0.135**	0.046	0.062	0.214***	0.192***	0.226***	-0.311***	-0.311***
Shares #	0.041	-0.056	0.104*	0.025	-0.156**	-0.061	0.081	-0.068	-0.008	0.010	0.0381
Loses temper	-0.169***	0.074	-0.010	0.017	**0.394*** **	0.039	-0.037	0.212***	0.067	-0.147**	-0.134**
Pref solitary	0.125*	0.089	-0.006	0.031	-0.076	0.155**	-0.032	0.006	0.194***	*-0.071*	-0.69
Well behaved #	0.250***	-0.041	0.029	0.035	-0.221***	-0.086	0.024	-0.242***	-0.008	*0.101**	0.061
Many worries	**0.399*** **	0.220***	0.175***	0.109*	-0.079	-0.002	0.229***	0.015	0.169***	-0.217***	-0.236***
Help others if hurt #	0.064	0.037	0.100*	0.106*	-0.087	-0.059	0.059	-0.131**	-0.050	0.009	0.016
Fidgeting	-0.075	0.038	0.012	0.001	0.092	0.117*	-0.036	0.136**	0.036	*-0.063*	-0.083
One good friend #	0.022	-0.079	0.026	0.007	-0.015	-0.060	-0.024	-0.008	-0.067	*0.056*	0.043
Often fights or bullies	-0.132**	-0.009	-0.099	-0.05	0.138	0.044	-0.059	0.033	-0.019	*0.027*	0.017
Often unhappy	0.255***	**0.400*** **	0.154**	0.177***	0.108*	0.054	0.140**	0.168**	0.210***	-0.319***	-0.321***
Generally liked by others #	0.045	-0.054	-0.000	0.063	-0.023	-0.059	0.057	-0.007	-0.032	0.022	0.019

Easily distracted	-0.062	0.076	-0.017	0.017	0.142**	0.260***	-0.063	0.142**	0.106*	-0.126*	-0.127*
Low confidence	**0.125***	0.068	0.055	-0.015	-0.024	0.025	0.039	0.033	0.144**	-0.083	-0.083
Kind to younger children #	0.065	0.015	0.028	0.007	-0.082	-0.068	0.050	-0.108*	-0.047	0.049	0.045
Often lies / cheats	-0.202***	-0.021	-0.062	-0.033	0.049	0.023	-0.132**	0.131**	0.010	0.001	0.014
Picked on or bullied	-0.030	0.111*	0.055	-0.023	0.013	0.071	-0.046	0.012	-0.032	*-0.029*	-0.044
Volunteers to help #	0.134**	0.022	0.031	0.082	-0.156**	-0.074	0.085	-0.086	-0.049	0.024	0.011
Thinks before acting #	0.138**	-0.035	0.067	0.103	-0.193***	-0.113*	0.055	-0.160**	-0.065	*0.072*	0.072
Steals	-0.126*	-0.054	-0.021	-0.031	0.004	0.007	-0.098	-0.014	-0.044	*0.053*	0.063
Better with adult than peers	0.121*	0.096	0.011	0.061	-0.017	0.116*	-0.044	-0.066	0.117*	*-0.083*	-0.087
Many fears	**0.340*****	0.199***	0.135**	0.049	-0.028	0.021	0.167***	0.019	0.133**	-0.201***	-0.218***
Good attention span / follow through #	0.057	-0.118*	0.009	-0.007	-0.169***	-0.446***	0.014	-0.227***	-0.164**	0.202***	0.221***

Notes: # Higher value is better. * P <0.05 **P<0.01 ***P<0.001. Bolded figures are those where CHU9D and SDQ have clear conceptual overlap according to Furber & Segal[12]. Italic figures in CHU9D adult weights column are the correlations where Furber & Segal[12] find correlations larger than 0.2 and where we find correlations of 0.1 or lower.

Wolf, RT., Ratcliffe, J., Chen, G., Jeppesen, P. The longitudinal validity of proxy-reported CHU9D.

Table C: Spearman rank correlation coefficients for CHU9D items and SDQ Impact items

	Worried	Sad	Pain	Tired	Annoyed	Schoolwork /homework	Sleep	Daily Routine	Activities	**CHU9D adult weights**	**CHU9D adolescent weights**
**Impact score**	0.183***	0.308***	0.074	0.113*	0.160**	0.248***	0.020	0.282***	0.346***	-0.330***	-0.329***
The difficulties upsets or distresses the child	0.237***	0.395***	0.149	0.182***	0.111*	0.024	0.087	0.176***	0.206***	-0.300***	-0.293***
Home life	0.049	0.217***	0.027	0.105*	0.248***	0.023	0.061	0.269***	0.072	-0.220***	-0.215***
Friendships	0.164**	0.201***	0.116	0.084	0.051	0.133**	0.050	0.113*	0.288***	-0.228***	-0.219***
Classroom learning	0.033	0.066	-0.063	-0.049	0.031	0.407***	-0.098	0.082	0.176***	-0.081	-0.110*
Leisure activities	0.106*	0.172***	0.0867	0.142**	0.108*	0.176***	0.063	0.181***	0.324***	-0.258***	-0.242***

Notes: * P <0.05 **P<0.01 ***P<0.001

Wolf, RT., Ratcliffe, J., Chen, G., Jeppesen, P. The longitudinal validity of proxy-reported CHU9D.

Table D: Spearman rank correlation coefficients for CHU9D items and KIDSCREEN-27 scales and items.

	Worried	Sad	Pain	Tired	Annoyed	Schoolwork /homework	Sleep	Daily Routine	Activities	**CHU9D adult weights**	**CHU9D adolescent weights**
**Physical Well-being**	-0.266***	-0.329***	-0.251***	-0.283***	-0.103*	-0.172***	-0.157**	-0.257***	-0.436***	0.438***	0.408***
General health status #	0.305***	0.318***	0.303***	0.120***	0.073	0.118*	0.146**	0.191***	0.334***	-0.373***	-0.324***
Feel fit and well	-0.347***	-0.361***	-0.287***	-0.267***	-0.120	-0.172***	-0.169***	-0.263***	-0.364***	0.450***	0.440***
Physically active	-0.138**	-0.199***	-0.156**	-0.191***	-0.034	-0.119*	-0.085	-0.176***	-0.352***	0.274***	0.245***
Run well	-0.125*	-0.212***	-0.187***	-0.180***	-0.037	-0.101*	-0.129**	-0.163***	-0.325***	0.303***	0.265***
Feelt energetic	-0.270***	-0.300***	-0.157**	-0.323***	-0.154**	-0.229***	-0.145**	-0.258***	-0.356***	0.410***	0.400***
**Psychological Well-being**	-0.439***	-0.541***	-0.186***	-0.309***	-0.222***	-0.265***	-0.220***	-0.334***	-0.430***	0.565***	0.571***
Life been enjoyable	-0.333***	-0.409***	-0.175***	-0.257***	-0.223***	-0.253***	-0.174***	-0.236***	-0.368***	0.443***	0.445***
In good mood	-0.341***	-0.397***	-0.196***	-0.254***	-0.251***	-0.167***	-0.187***	-0.260***	-0.331***	0.465***	0.453***
Have had fun	-0.292***	-0.384***	-0.104*	-0.184***	-0.183**	-0.203***	-0.129*	-0.252***	-0.360***	0.410***	0.404***
Felt sad #	0.0435***	**0.595*****	0.177***	0.238***	0.245***	0.205***	0.203***	0.280***	0.285***	-0.509***	-0.541***
Felt too sad and didn't want to do anything #	0.346***	0.411***	0.162**	0.288***	0.163**	0.245***	0.246***	0.304***	0.398***	-0.484***	-0.481***
Felt lonely #	0.313***	0.378***	0.087- 0.066	0.193***	0.062	0.221***	0.155**	0.185***	0.302***	-0.367***	-0.380***
Been happy with the way he/she is	-0.288***	-0.330***	-0.057	-0.201***	-0.063	-0.178***	-0.090*	-0.238***	-0.253***	0.316***	0.328***
**Autonomy & Parent Relation**	-0.004	-0.103*	-0.052	0.049	-0.177***	-0.153**	-0.079	-0.227***	-0.079	0.197***	0.200***
Child had enough time for him/herself	-0.085	-0.062	0.007	-0.042	-0.063	-0.025	-0.114*	-0.119**	0.028	0.106*	0.134**

Child been able to do the things that he/she wants to do in his/her free time	-0.093	-0.084	-0.055	-0.032	-0.057	-0.127*	-0.064	-0.153***	-0.093	0.151**	0.163**
Child felt that his/her parent(s) had enough time for him/her	-0.040	-0.147**	-0.047	-0.015	-0.152**	-0.067	-0.091	-0.125*	-0.047	0.178***	0.161**
Child felt that his/her parent(s) treated him/her fairly	0.056	-0.137**	-0.066	0.029	-0.319***	-0.035	-0.083	-0.239***	-0.031	0.189***	0.184***
Child been able to talk to his/her parent(s) when he/she wanted to	0.051	-0.102*	0.005	0.036	-0.159**	-0.073	-0.072	-0.192***	-0.076	0.176***	0.159**
Child had enough money to do the same things as his/her friends	0.057	0.040	-0.026	0.121*	0.007	-0.105*	0.022	-0.062	-0.029	0.016	0.022
Child felt that he/she had enough money for his/her expenses	0.087	0.050	-0.096	0.081	-0.026	-0.129*	0.036	-0.074	-0.033	0.018	0.019
**Social Support & Peers**	-0.150**	-0.173***	-0.079	-0.056	-0.028	-0.121*	-0.046	-0.117*	-0.316***	0.217***	0.187***

Child spent time with his/her Friends	-0.110*	-0.150**	-0.082	-0.128*	-0.033	-0.145**	-0.028	-0.120*	-0.334***	0.218***	0.184***
Child had fun with his/her friends	-0.188**	-0.164**	-0.031	-0.077	-0.014	-0.106*	-0.055	-0.108*	-0.321***	0.218***	0.184***
Child and his/her friends helped each other	-0.106*	-0.119*	-0.036	0.017	-0.020	-0.104*	-0.017	-0.063	-0.250***	0.146**	0.125*
Child been able to rely on his/her friends	-0.120*	-0.138**	-0.054	-0.010	-0.013	-0.076	-0.087	-0.094	-0.210***	0.161**	0.144**
**School Environment**	-0.114*	-168***	0.039	-0.012	-0.118*	-0.458***	-0.080	-0.199***	-0.297***	0.266***	0.260***
Child been happy at school	-0.301***	-0.263***	-0.054	-0.126*	-0.081	-0.347***	-0.145	-0.155**	-0.373***	0.328***	0.323***
Child got on well at school	-0.083	-0.092	0.062	0.035	-0.063	-0.497***	0.006	-0.187***	-0.269***	0.201***	0.208***
Child been able to pay attention	-0.056	-0.163**	0.027	-0.102*	-0.147**	-0.388***	-0.097	-0.188***	-0.252***	0.249***	0.244***
Child got along well with his/her teachers	0.054	-0.038	0.089	0.130**	-0.100*	-0.224***	-0.049	-0.084	-0.073	0.079	0.061

Notes: # Higher value is better. * P <0.05 **P<0.01 ***P<0.001
